# Predictive value of CAC score combined with clinical features for obstructive coronary heart disease on coronary computed tomography angiography: a machine learning method

**DOI:** 10.1186/s12872-022-03022-9

**Published:** 2022-12-26

**Authors:** Yongkui Ren, Yulin Li, Weili Pan, Da Yin, Jie Du

**Affiliations:** 1 Beijing Anzhen Hospital, Capital Medical University, No. 2 Anzhen Road, Chaoyang District, Beijing, China; 2 Key Laboratory of Remodeling-Related Cardiovascular Diseases, Ministry of Education, Beijing, China; 3 Beijing Institute of Heart, Lung, and Blood Vessel Disease, Beijing, China; 4 Department of Cardiology, Shenzhen People’s Hospital, 2nd Clinical Medical College of JINAN University, 1st Affiliated Hospital of Southern University of Science and Technology, ShenZhen, China; 5 Department of Cardiology, 1st Affiliated Hospital of Dalian Medical University, Dalian, China

**Keywords:** Machine learning, Random forest, Coronary artery calcification score, Obstructive coronary artery disease

## Abstract

**Objective:**

We investigated the predictive value of clinical factors combined with coronary artery calcium (CAC) score based on a machine learning method for obstructive coronary heart disease (CAD) on coronary computed tomography angiography (CCTA) in individuals with atypical chest pain.

**Methods:**

The study included data from 1,906 individuals undergoing CCTA and CAC scanning because of atypical chest pain and without evidence for the previous CAD. A total of 63 variables including traditional cardiovascular risk factors, CAC score, laboratory results, and imaging parameters were used to build the Random forests (RF) model. Among all the participants, 70% were randomly selected to train the models on which fivefold cross-validation was done and the remaining 30% were regarded as a validation set. The prediction performance of the RF model was compared with two traditional logistic regression (LR) models.

**Results:**

The incidence of obstructive CAD was 16.4%. The area under the receiver operator characteristic (ROC) for obstructive CAD of the RF model was 0.841 (95% CI 0.820–0.860), the CACS model was 0.746 (95% CI 0.722–0.769), and the clinical model was 0.810 (95% CI 0.788–0.831). The RF model was significantly superior to the other two models (*p* < 0.05). Furthermore, the calibration curve and Hosmer–Lemeshow test showed that the RF model had good classification performance (*p* = 0.556). CAC score, age, glucose, homocysteine, and neutrophil were the top five important variables in the RF model.

**Conclusion:**

RF model was superior to the traditional models in the prediction of obstructive CAD. In clinical practice, the RF model may improve risk stratification and optimize individual management.

**Supplementary Information:**

The online version contains supplementary material available at 10.1186/s12872-022-03022-9.

## Introduction

Individuals with atypical chest pain are common in clinical practice. It is a big challenge for cardiologists to more accurately distinguish patients with obstructive coronary artery disease (CAD) from atypical patients. Coronary computed tomography angiography (CCTA) can non-invasively evaluate the severity of CAD [1, 2]. However, intensive X-ray and contrast usage and high-cost limit wide applications of CCTA in routine screenings. Similarly, several guidelines have recommended using the Diamond and Forrester model (DF) or the Duke clinical score (DCS) to estimate the pretest probability of CAD in patients with chest pain [3, 4]. Nevertheless, recent studies have proved that DF and DCS score tend to overestimate the probability of obstructive CAD [5, 6]. Given the above, it is necessary to seek a new prediction model for obstructive CAD.

Coronary artery calcium (CAC), known as a biomarker of subclinical atherosclerosis, is tightly related to the occurrence of future cardiovascular events and all-cause mortality [7–10]. The addition of CAC scores to prediction models has been reported to enhance performance for obstructive CAD [11, 12].

Machine learning (ML) has emerged as a neoteric category of artificial intelligence and is widely applied to healthcare data analysis [13–15]. Random forest (RF), as a classic ML algorithm, is good at using out-of-bag estimates and an internal bootstrap to reduce and select predictive features and avoid over-fitting[16]. In this study, we sought to develop the RF model to predict patients with obstructive CAD and compare the prediction performance between the RF model and two traditional logistic regression (LR) models.

## Methods

### Study population

**Fig. 1:**
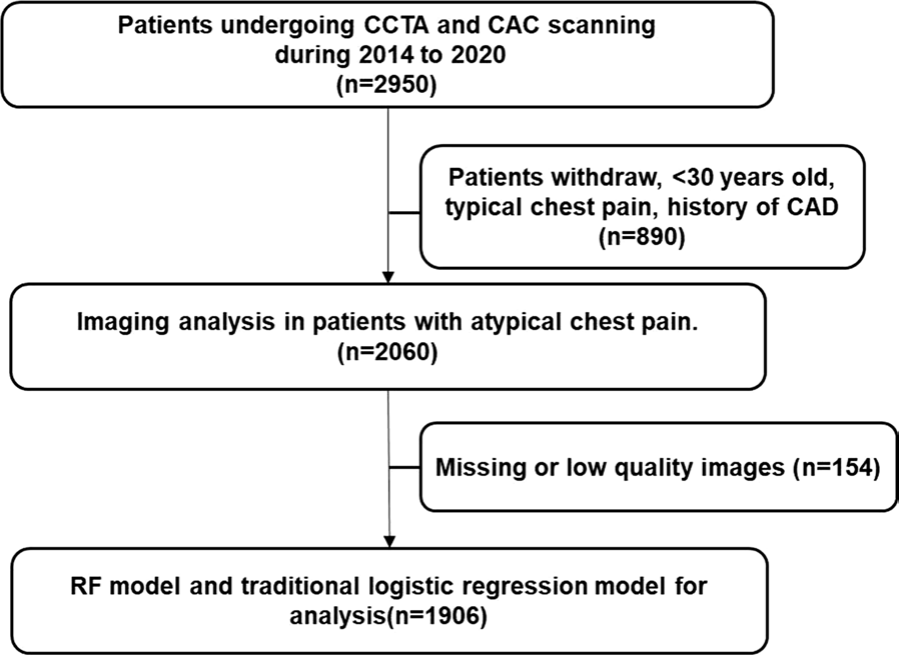
The flow chart of this research. CCTA, coronary computed tomography angiography; CAD, coronary artery disease; RF, random forest; CAC, coronary artery calcium

We retrospectively screened patients admitted to the First Affiliated Hospital of Dalian Medical University from January 2014 to December 2020 and the detailed flow chart was shown in Fig. 1. The data includes demographics, CAC scores, and clinical and imaging parameters. Inclusion criteria were patients with ≥ 30 years old, atypical chest pain, no history of CAD, and underwent CAC and CCTA scanning. Individuals with missing data of scan identifiers, no-dedicated CAC score, no-CAC scanning, uncertain date of birth, and uncertain data of scan were excluded. A total of 1,906 patients were enrolled in the study. This study was approved by the Ethics Committee Board of the First Affiliated Hospital of Dalian Medical University.

### Coronary computed tomography angiography and coronary artery calcium scanning

**Fig. 2:**
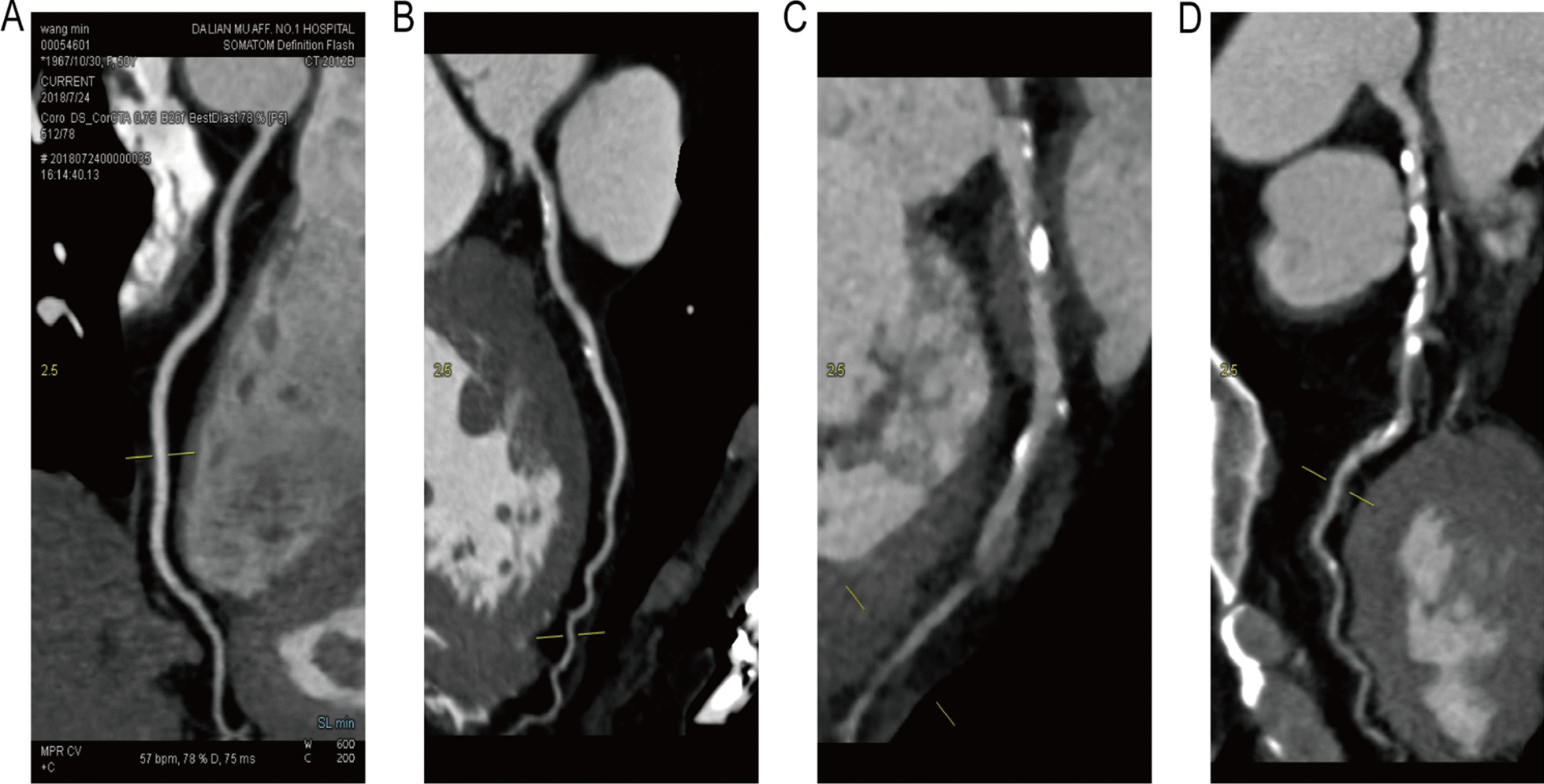
Representative images of CCTA of different levels of calcification. **A** Normal coronary without calcification. **B** Coronary with light calcification. **C** Coronary with moderate calcification. **D** Coronary with severe calcification. CCTA, coronary computed tomography angiography

The scanner (dual-source, Somatom Definition CT, Siemens, Erlangen, Germany) was applied to acquire and process the CCTA images and as well as CAC scores. All the processes strictly followed the guidelines recommended by the Society of Cardiovascular Computed Tomography [17]. Meanwhile, two independent and professional imaging physicians assessed all images, identified the severity of CAD, and determined a CAC score based on the Agatston method [18]. Furthermore, representative CCTA images of different levels of calcification were shown in Fig. 2. The presence of diameter stenosis ≥ 50% in any of the four major epicardial coronary arteries detected on CCTA was defined as obstructive CAD and the outcome of the present study.

### Building a machine learning model

A total of 63 available demographic and clinical variables of screened individuals were analyzed in this study, including age, sex, cardiovascular risk factors (the history of hypertension and diabetes mellitus, current smoker), baseline plasma lipid levels (total cholesterol, high-density lipoprotein cholesterol (HDL-C), low-density lipoprotein cholesterol (LDL-C) and triglyceride), electrocardiogram parameters, imaging parameters (ultrasonic cardiogram, carotid ultrasonography) and so on (shown in Additional file 3: Table S1).

RF, based on decision tree mechanisms, combines various decision tree classifiers to provide the final classification and improve classification accuracy. In this study, the RF model included all available variables, and all included individuals were randomly divided into the training set (70%) and the validation sets (30%). The random processes were continuously repeated until all the data equally distributed in both sets. Meanwhile, tuning was considered to avoid overfitting for ML-based models and the optimal hyper-parameter in the training process for ML models was fivefold cross-validation.

### Comparison of predictive performance between the RF model and LR models

To evaluate the predictive performance and clinical value of our proposed RF model, we compared the RF model with two traditional Logistic Regression (LR) models which were constructed as follows: (1) a model (regarded as CACS model) trained with CAC score alone; and (2) the other model (considered as a clinical model) trained with cardiovascular risk factors (age, sex, the history of hypertension and diabetes mellitus, current smoker, total cholesterol, LDL-C, HDL-C, and CAC score. To make paired comparisons, the same folds and cross-validation procedures were performed in the training and evaluation of the two LR models as the RF model.

### Statistical analysis

Continuous variables are reported as mean ± SD for normally distributed variables or median (interquartile range) for non-normally distributed data and were compared with one-way ANOVA or non-parametric test. Categorical variables are expressed as a number (percentage) and were compared with chi-square tests or Fisher’s exact test.

The RF model was compared with the traditional LR models using the calibration curve and Hosmer–Lemeshow test. The area under the receiver operator characteristics curve (AUC) was applied to assess the performance of predictive models. To examine the added improvement of the RF model in predicting obstructive CAD, we utilized the continuous net reclassification index (NRI) and integrated discrimination improvement (IDI) analysis. The decision curve analysis (DCA) was used to compare the benefit of the three models at different threshold probabilities. Finally, The Youden index was also provided to summarize performance predictions. All statistical analyses were performed by R software (Version 4.0.3 R Foundation for Statistical Computing, Vienna, Austria). The R packages “caret,” “e1071,” “random-forest,” “dplyr,” “nricens,” “rmda,” “GLM,” “pROC” were used in this study. Statistical significance was defined as a *p* < 0.05 on a 2-tailed test.

## Results

### Demographic features

A total of 1,906 patients were finally included in the study. The occurrence of obstructive CAD was 16.4% (313 out of 1,906) within the studied cohort (as shown in Table 1; Additional file 4: Table S2). The mean age of the cohort was 57.4 years, 54.6% were men, 48.0% had hypertension, 26.1% had diabetes mellitus, and 28.9% were current smokers. The proportion of CAC score = 0 in this study was 42.4%, and the proportion of severe calcification (CAC score ≥ 400) was 11.4%.

**Table 1:** Baseline characteristics of the study population

	Total (n = 1906)
Age, years	57.4 ± 14.53
Male, n (%)	1041 (54.6)
Hypertention, n (%)	914 (48.0)
Diabetes mellitus, n (%)	498 (26.1)
Current smoker, n (%)	551 (28.9)
TC (mmol/L)	4.79 ± 1.05
TG (mmol/L)	1.40 (1.02–2.03)
HDL-C (mmol/L)	1.10 (0.93–1.31)
LDL-C (mmol/L)	2.63 ± 0.72
Serum creatinine, (µmol/L)	65.0 (54.0–78.0)
LVEF, %	57.81 ± 4.92
OCAD, n (%)	313 (16.4)
CACS, AU	3.35 (0.00–109.63)
*CACS (n = 1906)*	
0	850 (44.5)
1–99	563 (29.5)
100–399	275 (14.4)
≥ 400	218 (11.4)

Values are presented as mean ± SD, median (25th–75th percentiles) or n (%)

TC, total cholesterol; TG, triglyceride; HDL-C, high density lipoprotein cholesterol; LDL-C, low density lipoprotein cholesterol; LVEF, left ventricular ejection fraction; CACS, coronary artery calcium score; OCAD, obstructive coronary artery disease

### Comparison of predictive performance for obstructive CAD between the RF model and traditional LR models

**Fig. 3:**
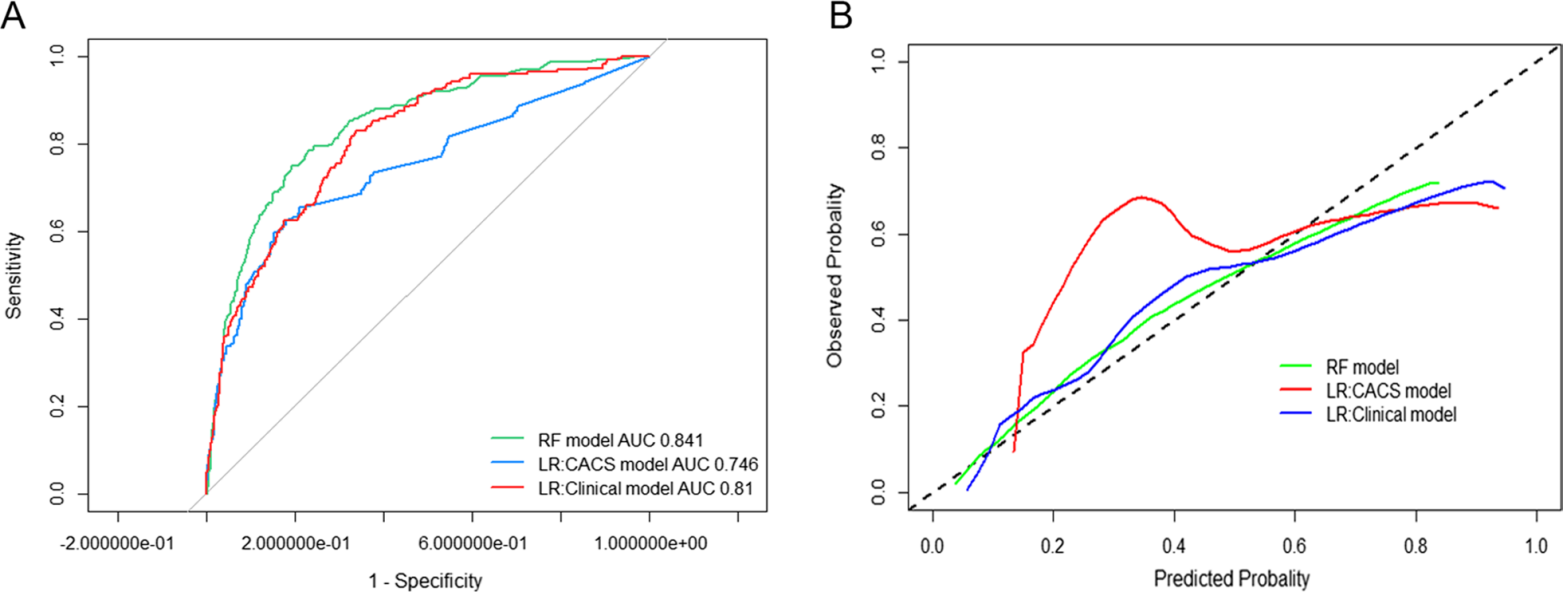
AUC (**A**) and calibration curve (**B**) of the different models for the prediction of obstructive coronary artery disease on CCTA. CACS model including CAC score only; the clinical model includes atherosclerotic cardiovascular disease risk factors and CAC score. AUC, the area under the receiver operating characteristics curve; CCTA, coronary computed tomography angiography; LR, logistic regression; RF, random forest; CAC, coronary artery calcium

To avoid overfitting of the model, we explored correlation coefficients between variables and showed them as a matrix in Additional file 1: Figure S1. Furthermore, within the test set, the predictive performances of the three models were compared and detailed in Fig. 3A, which were evaluated based on the area under the receiver operating characteristics curve (area under AUC). Interestingly, the RF model produced the best performance in terms of predicting individuals with obstructive CAD, with an AUC of 0.841 (95% CI 0.820–0.860) compared with the CACS model (AUC 0.746, 95% CI 0.722–0.769), and clinical model (AUC 0.810, 95% CI 0.788–0.831), *p* < 0.05 for all comparisons.

Model calibration was deployed to evaluate the possibility of a given new observation belonging to each of the already established classes (the presence or absence of CAD on CCTA). RF model indicated a minimal difference between the predicted and observed probability of obstructive CAD. Therefore, the RF model achieved a good model fit (as shown in Fig. 3B). Furthermore, the Hosmer–Lemeshow test indicated that the RF model had a high calibration (*p* = 0.556), while the other two models were disappointing (*p* < 0.05). Additionally, continuous NRI was 0.18 (95% CI 0.040–0.327), and IDI was 0.03 (95% CI 0.005–0. 058) when the RF model was compared with the clinical model (as shown in Table 2), with *p* < 0.05 for all comparisons.

**Table 2:** The comparisons of NRI and IDI between the different models

	NRI (95%CI)	*P*	IDI (95%CI)	*P*
RF model versus clinical model	0.18 (0.040–0.327)	0.012	0.03 (0.005–0.059)	0.021
Events	0.08 (− 0.053 to 0.221)	0.218		
Non-events	0.09 (0.035–0.158)	0.001		
RF model versus CACS model	0.82 (0.696–0.953)	< 0.0001	0.13 (0.10–0.155)	< 0.0001
Events	0.47 (0.352–0.578)	< 0.0001		
Non-events	0.35 (0.298–0.406)	< 0.0001		
Clinical model versus CACS model	0.64 (0.501–0.767)	< 0.0001	0.09 (0.075–0.117)	< 0.0001
Events	0.28 (0.153–0.393)	< 0.0001		
Non-events	0.36 (0.303–0.412)	< 0.0001		

CACS model including CAC score. Clinical model including ASCVD risk factors and CAC score

ASCVD, atherosclerotic cardiovascular disease; LR, logistic regression; RF, random forest; NRI

Table 3 showed the Youden index for each model, with our RF model having the greatest Youden index for obstructive CAD. At the optimal cutoff, we observed 74.9% sensitivity, 80.6% specificity, 43.6% positive predictive value, 94.1%negative predictive value, and 77.8% accuracy. Moreover, the RF model still displayed strong predictive capabilities in individuals stratified by age and sex (as shown the Additional file 2: Figure S2).

**Table3:** Optimal cutpoints and youden index for each model

	Cutpoint	SE	SP	PPV	NPV	Accuracy	Youden index
RF model	0.229	0.749	0.806	0.436	0.941	0.778	0.555
CACS model	0.136	0.655	0.789	0.384	0.919	0.722	0.444
Clinical model	0.129	0.825	0.644	0.317	0.948	0.734	0.468

SE, sensitivity; SP, specificity; PPV, positive predictive value; NPV, negative predictive value; RF, random forest; CACS model, CAC score only; clinical model, atherosclerotic cardiovascular disease risk factors and CAC score

### The relative importance of variables in RF algorithm

**Fig. 4:**
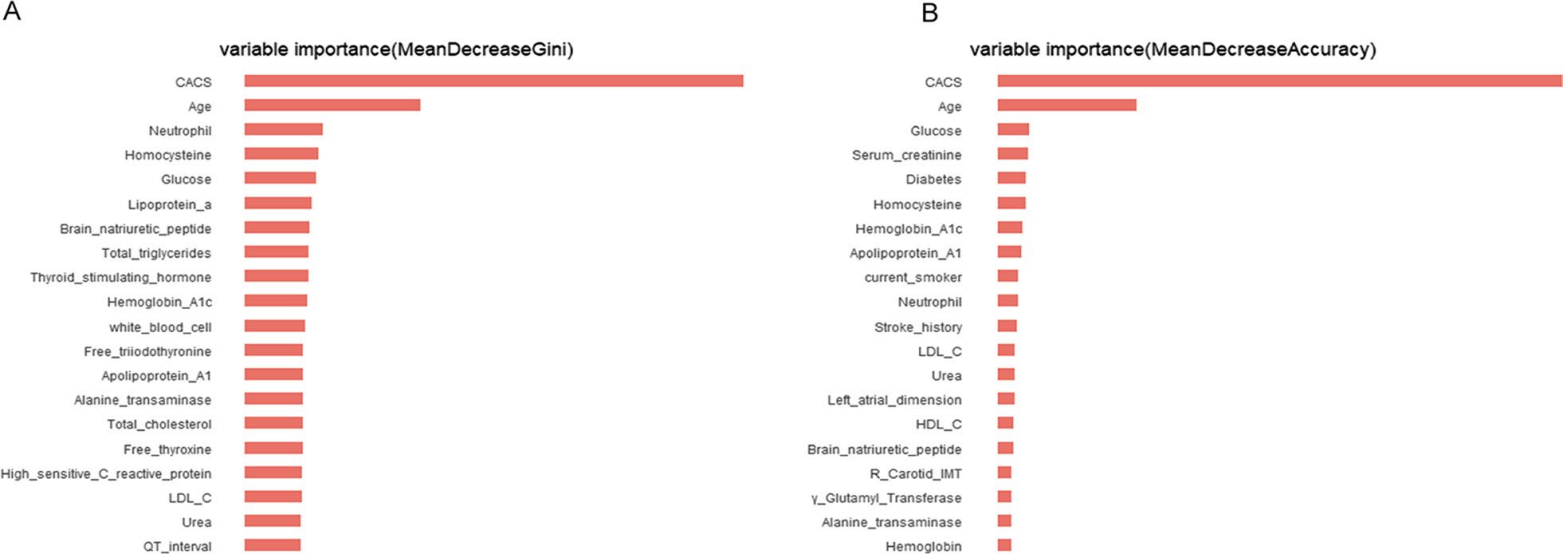
The importance of variables in the RF algorithm based on mean decrease Gini (**A**) and mean decrease accuracy (**B**). RF, random forest; CACS, coronary artery calcium score; LDL-C, low-density lipoprotein cholesterol; HDL-C, high-density lipoprotein cholesterol

We selected the variables that rank high in both mean decrease Gini and mean decrease accuracy. As shown in Fig. 4, the probability of the prevalence of obstructive CAD increased, with CACS increasing. That is, CACS had the highest predictive value for the presence of obstructive CAD. Moreover, the most predictive features (after the CAC score itself) were age and fasting glucose levels followed by plasma homocysteine levels and the number of neutrophils. Intriguingly, left atrial dimension, carotid intima-media thickness (IMT) and QT interval might also have positive effects on the incidence of obstructive CAD among imaging parameters.

### The DCA of the three prediction models

**Fig. 5:**
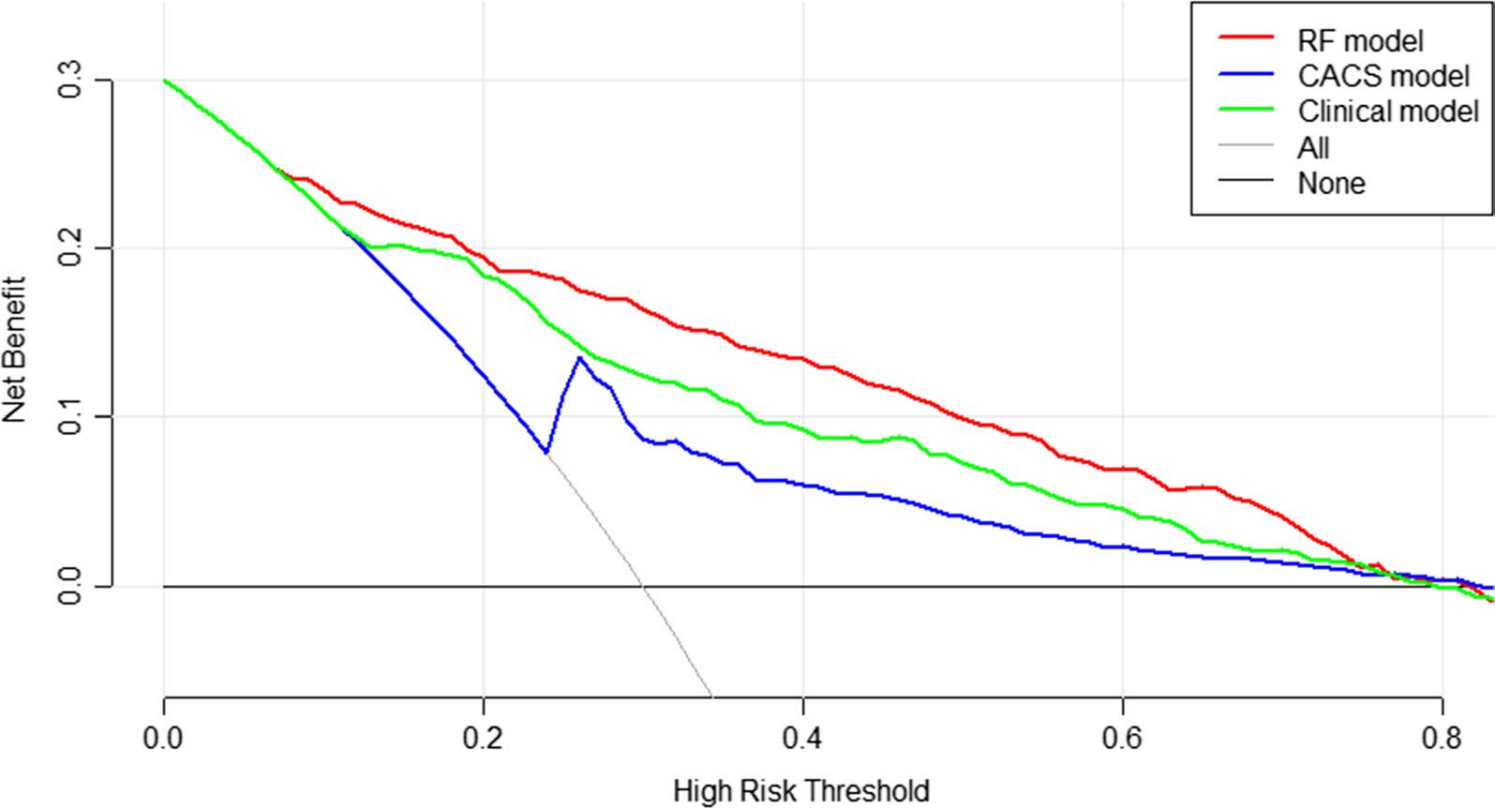
The decision curve analysis (DCA) of the three prediction models. Theoretically, all patients without obstructive coronary artery disease are represented by a black line, and all patients with obstructive coronary artery disease are represented by a gray line. The x-axis presented the Threshold probability. CACS model including CAC score only; The clinical model including atherosclerotic cardiovascular disease risk factors and CAC score

The DCA was used to compare the benefit of the RF model, CACS model, and Clinical model. We found that if the threshold probability in the clinical decision was > 10%, the patients would benefit more from the RF model than either the CACS model or the Clinical model (as shown in Fig. 5).

## Discussion

In this study, we have shown that the RF model integrating clinical variables and CAC score can obtain superior prognostic performance than the traditional LR models for obstructive CAD on CCTA. In addition, our comprehensive RF model obtained high concordance between the predicted risk and actual observed risk. CAC score was the most important variable in the RF model, followed by age, fasting glucose levels, plasma homocysteine levels, and the number of neutrophils.

Obstructive CAD is the most common etiology of atypical chest pain, which significantly increase mortality and healthcare expenditure. To noninvasively predict the occurrence of CAD, many models have been developed, such as CCTA, CACS, and cardiac magnetic resonance angiography [3, 4]. Nevertheless, the performance of many existent models is limited in the presence of obstructive CAD [19, 20]. Beyond that, the discriminative ability of some models has become lower in more than one external population in an ever-changing world [6]. Therefore, there is an urgent need for optimal predictive models for obstructive CAD in individuals with atypical chest pain.

ML, as a scientific algorithm, can make data-driven predictions by learning from the training set and finishing subsequent prediction tasks in an independent set [21]. Compared with other ML algorithms, such as neural network (NNET) and support vector machine (SVM), RF does not need to select features in advance and prevent over-fitting [16, 22]; Compared with the traditional LR models, RF, a classic ML algorithm, can account for non-linear and higher dimensional relationships between a multitude of variables that could potentially lead to an improved explanatory model. Similarly, our research found that although the LR models containing the CAC score have moderate predictive power, however, the calibration curve fitting did not achieve well which has been proved by the Hosmer–Lemeshow. On the contrary, the RF model showed a better predictive performance for obstructive CAD. Additionally, RF models have shown equal or better performance than humans in medical practices such as diagnosis, decision-making, and risk prediction in cardiology [16]. Our findings uphold the RF model based on all available information and CAC scores can more accurately identify high-risk individuals and improve the clinical use of the CAC scanning in risk assessment and guiding management decisions [11, 23–25].

In the order of variable importance, consistent with the previous studies, the CAC score is superior to traditional cardiovascular risk factors, such as age, sex, smoking, the presence of diabetes mellitus and hyperlipidemia, and so on. The CAC score measured by non-contrast cardiac-gated computed tomography (CT) provides an evaluation of the global burden of coronary atherosclerosis. Furthermore, the CAC score can provide a long-term and independent prognosis for the clinical risk of cardiovascular disease (CVD) and CAD events [7–9]. Therefore, accurate coronary calcification detection and assessment can aid in clinical decision-making. Recent research has shown that deep learning techniques, irrespective of picture quality and calcification, can precisely estimate coronary artery calcification from CT angiography images [15, 26]. In future clinical applications, it might have a significant impact.

A good model should take into account not only its diagnostic effectiveness but also its repeatability, noninvasiveness, and simplicity. In our study, other CT variables such as the total number of calcified coronary lesions, plaque density, the presence of thoracic aorta calcification, and so on, which have been revealed to increase the predictive potency of CAC for CVD events were not included in the present prediction model[27, 28]. However, the prediction results of the RF model in our investigation were similar to those of the previously reported Extreme Gradient Boosting (XGBoost) model [11]. Additionally, our preliminary experiments showed that the RF model had better calibration than XGBoost. Considering its effect on the insensitivity of missing values and the advantages of dealing with high-dimensional data make it easier to generalize in clinical practice. Last but not least, current guidelines have recommended that CACS can be used to guide preventive therapies in asymptomatic individuals at intermediate risk for CVD events [29, 30]. Given the above, patients at lower risk in the RF model may not require further testing, such as CCTA or Coronary angiography.

Several limitations of the present study should be paid more attention to. Firstly, the present investigation was lack of external validation in an independent cohort, which was planned for subsequent analysis. Secondly, the presence of severe calcification may lead to overestimates % stenosis on CCTA. Hence, more than 50% stenosis on CCTA may not represent the accuracy > 50% stenosis evaluated by coronary angiography. Thirdly, further study with long follow-up times is very necessary to assess the long-term predictive role of the CAC score. Fourthly, all the screened subjects were all from China, thus, the predictive model may be not suitable for other ethnic groups. Finally, we did not use multiple ML algorithms for this research, but the RF model has shown better predictive ability in previous studies.

## Conclusion

The predictive performance of the RF model integrating clinical variables and CAC score is superior to models combining traditional risks and CAC score for the presence of obstructive CAD in patients with atypical chest pain. It may be unreasonable for individuals at a low risk assessed by the prediction model to receive a further invasive examination.

## Supplementary Information

**Additional file 1.** **Supplementary Figure 1**. **A matrix diagram of several variables**. Red means positive correlation, blue means negative correlation. LDL-C, low-density lipoprotein cholesterol; HDL-C, high-density lipoprotein cholesterol.

**Additional file 2.** **Supplementary Figure 2**. **Subgroup analysis of different prediction models**. CACS model including CAC score only. Clinical model including atherosclerotic cardiovascular disease risk factors and CAC score. AUC, area under the receiver operating characteristics curve; LR, Logistic Regression; RF, Random Forest; CAC, coronary artery calcium.

**Additional file 3.** **Supplementary Table 1**. Pre-implant clinical features included in the analysis.

**Additional file 4.** **Supplementary Table 2**. Comparasions bwtween the test cohort and the training cohort.

## Data Availability

The data are available from the corresponding authors upon reasonable request.
