# Supplementary figures and images for: Predictive value of CAC score combined with clinical features for obstructive coronary heart disease on coronary computed tomography angiography: a machine learning method
Source: BMC Cardiovasc Disord. 2022 Dec 26;22:569. doi: 10.1186/s12872-022-03022-9 (PMC9793556; doi:10.1186/s12872-022-03022-9)

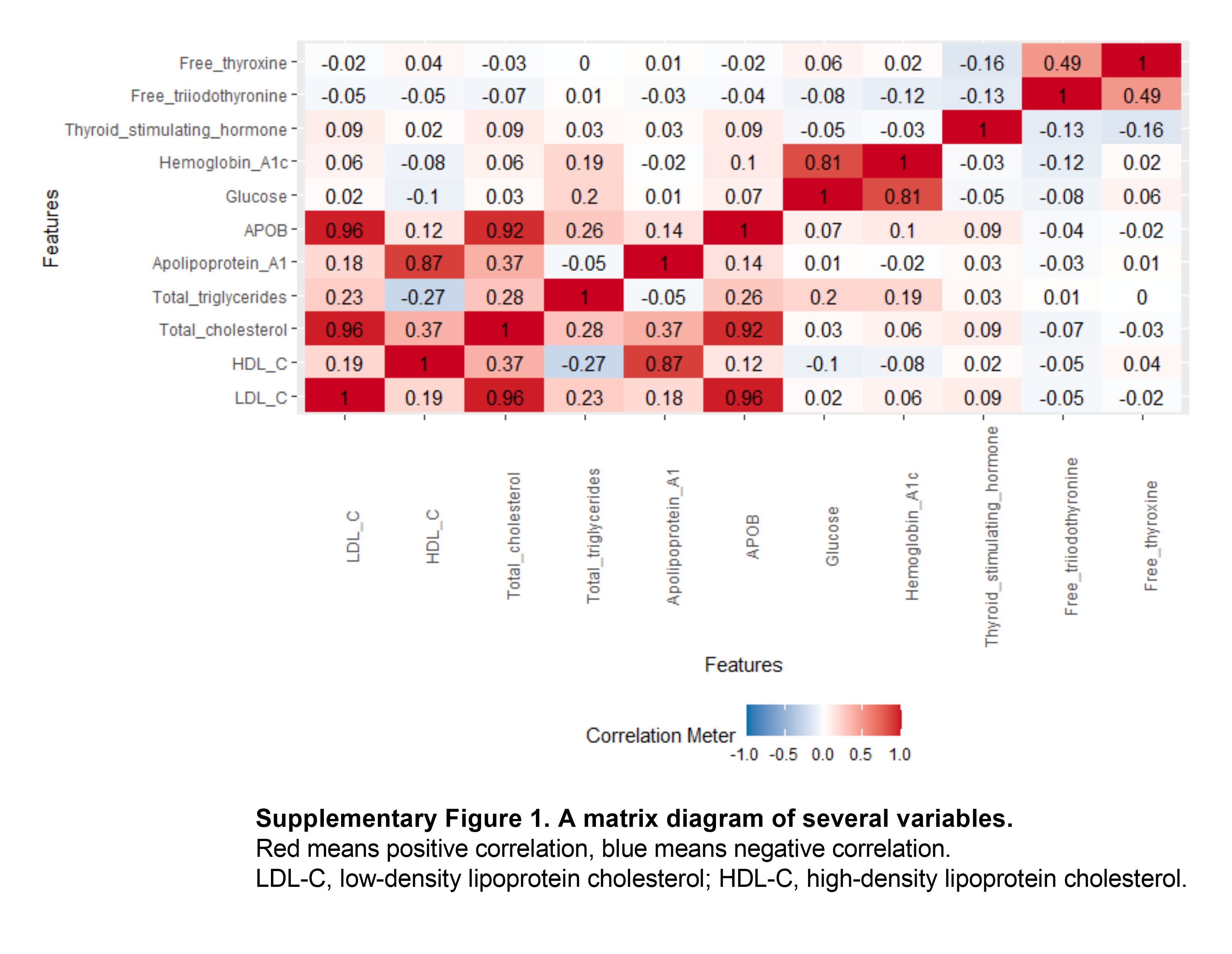

Supplement: Supplementary file 1 — Additional file 1. Supplementary Figure 1. A matrix diagram of several variables. Red means positive correlation, blue means negative correlation. LDL-C, low-density lipoprotein cholesterol; HDL-C, high-density lipoprotein cholesterol. [file 12872_2022_3022_MOESM1_ESM.tif]

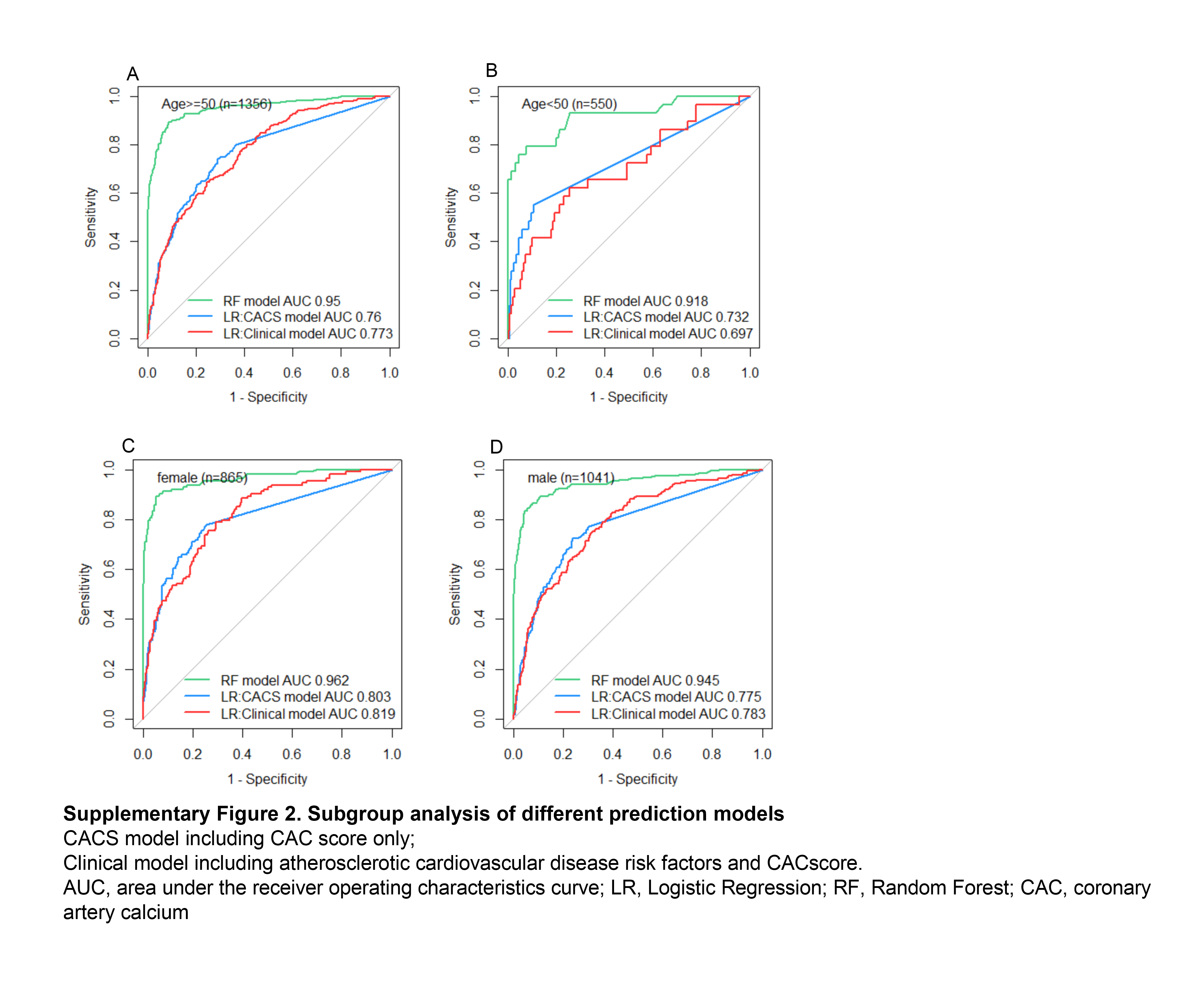

Supplement: Supplementary file 2 — Additional file 2. Supplementary Figure 2. Subgroup analysis of different prediction models. CACS model including CAC score only. Clinical model including atherosclerotic cardiovascular disease risk factors and CAC score. AUC, area under the receiver operating characteristics curve; LR, Logistic Regression; RF, Random Forest; CAC, coronary artery calcium. [file 12872_2022_3022_MOESM2_ESM.tif]
