# Supplementary material for: Predictive value of CAC score combined with clinical features for obstructive coronary heart disease on coronary computed tomography angiography: a machine learning method
Source: BMC Cardiovasc Disord. 2022 Dec 26;22:569. doi: 10.1186/s12872-022-03022-9 (PMC9793556; doi:10.1186/s12872-022-03022-9)
Supplement: Supplementary file 3 — Additional file 3. Supplementary Table 1. Pre-implant clinical features included in the analysis. [file 12872_2022_3022_MOESM3_ESM.docx]

**Supplementary Table 1** Pre-implant clinical features included in the analysis

| **Demographics** | **Comorbidities** | **ECG/** **imaging parameters** | **Laboratory Results** | |
| --- | --- | --- | --- | --- |
| age  gender  height  weight  SBP | hypertension  diabetes mellitus  stroke  atrial fibrillation  tumor  OSA  PAD | Heart rate  P-R interval.  QRS duration  QT Interval  Corrected QT Interval  Left atrial diameter  right atrial diameter  right ventricular diameter  Left Ventricular Hypertrophy  LVEF  [LVDD](http://abbr.dict.cn/left%20ventricular%20diastolic%20dimension/lvdd)  IVST  E/A ratio  E/e'  R-Carotid-IMT  L-Carotid-IMT  CACS | white blood cell  haemoglobin  platelets  neutrophils  hs-CRP  NT-proBNP  D-dimer  creatine kinase  creatine kinase isoenzyme  Troponin I  total cholesterol  triglycerides  HDL-C  LDL-C  Alkaline Phosphatase  Aspartate Aminotransferase | alanine aminotransferase  γ-Glutamyltransferase  Homocysteine  lipoprotein a  Apolipoprotein AI  Apolipoprotein B  Glucose  hemoglobin A1c  serum creatinine  serum uric acid  serum urea  TSH  Free Triiodothyronine  free thyroxine  serum sodium  serum potassium |
| SBP, systolic blood pressure; OSA, Obstructive Sleep Apnea; LVEF, left ventricular ejection fraction; LVDD, left ventricular end diastolic dimension; IVST, interventricular septal thickness; E/A ratio, the ratio of E to mitral peak velocity of late filling; E/e', the ratio of mitral peak velocity of early filling to early diastolic mitral annular velocity;IMT, intima-media thickness;CACS, Coronary artery calcium score; hs-CRP, high-sensitivity C-reactive protein; HDL-C, high-density lipoprotein cholesterol; LDL-C, low-density lipoprotein cholesterol; TSH, thyroid-stimulating hormone. | | | | |
