# Supplementary material for: Predictive value of CAC score combined with clinical features for obstructive coronary heart disease on coronary computed tomography angiography: a machine learning method
Source: BMC Cardiovasc Disord. 2022 Dec 26;22:569. doi: 10.1186/s12872-022-03022-9 (PMC9793556; doi:10.1186/s12872-022-03022-9)
Supplement: Supplementary file 4 — Additional file 4. Supplementary Table 2. Comparasions bwtween the test cohort and the training cohort. [file 12872_2022_3022_MOESM4_ESM.docx]

| **Supplementary Table 2. Comparasions bwtween the test cohort and the training cohort.** | | | | |
| --- | --- | --- | --- | --- |
|  | ALL  (N=1906) | Test Cohort  (N=572) | Training Cohort  (N=1334) | *p* |
| Age, y | 57.4 (14.5) | 57.0 (14.5) | 57.6 (14.5) | 0.431 |
| Male, n (%) | 1041 (54.6%) | 311 (54.4%) | 730 (54.7%) | 0.927 |
| Hypertention, n (%) | 914 (48.0%) | 267 (46.7%) | 647 (48.5%) | 0.497 |
| Diabetes mellitus, n (%) | 498 (26.1%) | 143 (25.0%) | 355 (26.6%) | 0.498 |
| Current smoker, n (%) | 551 (28.9%) | 165 (28.8%) | 386 (28.9%) | 1.000 |
| TC (mmol/L) | 4.79 (1.05) | 4.83(1.04) | 4.77(1.05) | 0.269 |
| TG (mmol/L) | 1.40 (1.03,2.03) | 1.38 (1.05,2.01) | 1.41 (1.02,2.04) | 0.912 |
| HDL-C(mmol/L) | 1.10 (0.93,1.31) | 1.08 (0.93,1.29) | 1.11 (0.92,1.31) | 0.598 |
| LDL-C(mmol/L) | 2.63 (0.72) | 2.67 (0.72) | 2.61 (0.73) | 0.131 |
| Creatinine, (umol/L) | 65.0 (54.0,78.0) | 65.0 (54.0,77.0) | 66.0 (54.0,78.0) | 0.232 |
| LVEF, % | 57.8 (4.92) | 57.5 (5.50) | 57.9 (4.65) | 0.140 |
| OCAD, n (%) | 313 (16.4%) | 90 (15.7%) | 223 (16.7%) | 0.643 |
| CACS, AU | 3.35 (0.00,109) | 2.17 (0.00,94.7) | 4.35 (0.00,117) | 0.91 |
| CACS (n=1906) |  |  |  | 0.600 |
| 0 | 809 (42.4%) | 239 (41.8%) | 570 (42.7%) |  |
| 1-99 | 604 (31.7%) | 191 (33.4%) | 413 (31.0%) |  |
| 100-399 | 275 (14.4%) | 75 (13.1%) | 200 (15.0%) |  |
| ≥400 | 218 (11.4%) | 67 (11.7%) | 151 (11.3%) |  |
| Values are presented as mean ± SD, median (25th–75th percentiles) or n (%).  TC, total cholesterol, TG, triglyceride, HDL-C, high density lipoprotein cholesterol, LDL-C, low density lipoprotein cholesterol, LVEF, left ventricular ejection fraction , CACS, coronary artery calcium score. OCAD , obstructive coronary artery disease | | | | |
